# Evaluating Gyratory Compaction Characteristics of Unbound Permeable Aggregate Base Materials from Meso-Scale Particle Movement Measured by Smart Sensing Technology

**DOI:** 10.3390/ma14154287

**Published:** 2021-07-31

**Authors:** Yuanjie Xiao, Meng Wang, Xiaoming Wang, Juanjuan Ren, Weidong Wang, Xiaobin Chen

**Affiliations:** 1 School of Civil Engineering, Central South University, Changsha 410075, China; menghm@csu.edu.cn (M.W.); Xiaoming-Wang@csu.edu.cn (X.W.); 147745@163.com (W.W.); xiaobin_chen@csu.edu.cn (X.C.); 2 Ministry of Education (MOE) Key Laboratory of Engineering Structures of Heavy Haul Railway, Central South University, Changsha 410075, China; 3 School of Civil Engineering, Southwest Jiaotong University, Chengdu 610031, China

**Keywords:** gyratory compaction, particle movement, unbound permeable aggregate, particle shape, particle abrasion and breakage

## Abstract

The quality of compaction of unbound aggregate materials with permeable gradation plays a vital role in their field performance; however, there are currently few unanimously accepted techniques or quality control criteria available for ensuring adequate compaction of such materials in either laboratory or field applications. This paper presented testing results of a laboratory gyratory compaction study where the combinations of gyratory parameters were properly designed using the orthogonal array theory. Innovative real-time particle motion sensors were employed to record particle movement characteristics during the compaction process and provide a meso-scale explanation about compaction mechanisms. Particle abrasion and breakage were also quantified from particle shape digitized from the three-dimensional (3D) laser scanner before and after compaction. The optimal combination of gyratory parameters that yields the best compaction performance was determined from the orthogonal testing results with the relative importance of major influencing parameters ranked accordingly. Meso-scale particle movement at the upper center and center side positions of the specimen are promising indicators of compaction quality. The gyratory compaction process can be consistently divided into three distinct stages according to both macro-scale performance indicators and meso-scale particle movement characteristics. A statistically significant bi-linear relationship was found to exist between relative breakage index and maximum abrasion depth, whereas the quality of compaction and the extent of particle breakage appear to be positively correlated, thus necessitating the cost-effective balance between them. The results of this study could provide technical insights and guidance to field compaction of unbound permeable aggregates.

## 1. Introduction

Compaction is the most cost-effective means to improve mechanical properties, drainability, and long-term durability (e.g., wet–dry and freeze–thaw) of earth materials such as fill materials used in roadway, railway, and airfield foundations [1,2,3]. Inadequate compaction, among many others, is generally attributable to structural failure and settlement problems under actual traffic. Impact compaction (e.g., standard and modified Proctor tests) and vibratory compaction (e.g., the ASTM D 4253 test procedure) are probably the most commonly used laboratory soil compaction procedures, while a vibratory compactor is used in most cases of field compaction. A series of studies have been conducted on compaction mechanisms, mainly focused on the comparison among different compaction methods, factors influencing compaction, and macro-scale indices (e.g., dry density, moisture content, and degree of compaction) for compaction quality assessment [4,5,6]^.^ The results of those studies show that particle size distribution (or gradation) and moisture content have important effects on the achieved dry density of compacted soils, which in turn affects permeability, strength, resilient modulus, and permanent deformation behavior significantly. Pawel et al. [7] found that a “locking point” exists during the impact compaction. Du et al. [8] investigated key parameters of vibration compaction for cement-treated aggregate mixture (CTAM). The different stages of vibratory compaction were identified by Hu [9]. The downfall of both impact and vibratory compaction methods includes the lack of resemblance to any type of field compaction, as well as the removal of larger-sized particles as limited by the mold size for minimizing size effects. Therefore, an additional rational and suitable compaction test procedure is needed to better simulate the loading conditions experienced during actual field compaction and traffic so as to yield laboratory densities no less than those achieved during field compaction.

The gyratory compaction method has emerged as an effective means for compacting soils and recycled granular materials, particularly for cohesionless soils such as sand and gravel. In addition to the relatively large specimen dimensions of, for instance, 150 mm diameter and 150 mm height, commercially available gyratory compactors can simultaneously apply a vertical load and a self-adjusting kneading action, and provide more comprehensive information than impact or vibratory compaction [10,11,12,13]. The dry unit weight of stabilized reclaimed materials obtained from the gyratory compaction with 500 kPa vertical pressure and 250 gyrations was found to be similar to that from standard Proctor compaction [14]. For fine-sand and silty-sand materials, Ping et al. [15] reported that gyratory compaction is the most suitable technique to simulate field compaction of granular soils, and thus can produce an internal structure of specimens that is of closer resemblance to that by actual field compaction and traffic [11]. The density and strength of the compacted gyratory specimen were closer to field measurements than the vibratory compaction [16]. Li et al. [17] obtained the relationship between moisture content-dry density-shear resistance-compaction energy of a large number of conventional and recycled roadbed fillers by installing a pressure distribution analyzer (PDA) on the rotary compaction equipment. However, the feasibility of gyratory compaction to relatively permeable unbound aggregates still remains unclear and thus needs to be confirmed.

The performance of unbound permeable aggregate base (UPAB) materials is affected by many factors such as the type, gradation, morphology, and compaction level of such materials [18,19,20]. The compaction promotes particle movement and rearrangement for improved inter-particle packing, and induces particle abrasion and breakage and the generation of finer fractions filling the voids of coarse particles [21,22,23]. The aggregate skeleton and internal structure of coarse-grained fill materials depends on the orientation, movement, and inter-particle contact and interlocking behavior of coarse aggregate particles, and is closely related to structural integrity, deformation resistance, and in-service performance. The aggregate skeleton in turn results from the movements of coarse particles and is affected by morphological characteristics (i.e., size and shape) of coarse particles. In essence, the compaction process is the one where coarse and fine fractions move relative to each other under external excitations until reaching a self-balance state with the densest possible packing; therefore, external compaction characteristics such as achieved dry density and specimen height reduction manifest internal particle contact and movement under compaction [24]. Nevertheless, the inherent mechanisms of internal structure formation and the underlying linkage between macro-scale compaction characteristics and meso-scale particle contact and movement are still not clear or understood adequately. The meso-scale evaluation of the compaction process from the perspective of individual particles is the key to better study the final structure and performance of coarse-grained fill materials. Previous studies have focused on studying the microstructure of earth materials and its relation with in-service performance by means of image-aided technologies, computed tomography (CT) scanning, and discrete element method (DEM) simulations. Gao et al. [25] found that the rotations of the coarse aggregates increase with compaction duration, which indicates that particle movement affects the degree of compaction. Li et al. [26] tracked the movements of coarse aggregates during the compaction by capturing marked aggregates (i.e., 19.0 mm particles inlaid with iron wire and 13.2 mm particles coated with iron powder) from CT scanning. Huang et al. [27] developed a new type of smart particulate sensor (“SmartRock”); tracked ballast particle movement under clean site and mud spot site; and reported that ballast particle movement is affected by many factors including ballast condition, train speed, and wheel load. Liu et al. [28] and Wang et al. [29] measured the translational and rotational movements of particles during the compaction process of asphalt mixture, where a smart sensor or wireless device was used to simulate the mineral aggregate. They concluded that monitoring the movements of coarse aggregates during the early compaction process is important for achieving good compaction workability of asphalt mixture. However, the relation between macro-scale compaction properties and meso-scale movements of differently sized particles for coarse-grained fill materials still needs to be examined integrally; further, the effects of major factors on the compaction of coarse-grained fill materials should also be examined systematically under this multi-scale framework in order to determine a practical combination of factors that produces optimum compaction behavior.

This paper aimed to explore the potential use of the gyratory compaction method as an effective means to compact UPAB materials and evaluate gyratory compaction characteristic from meso-scale particle movement. The workability index (K1) was calculated from the gyratory compaction curves to quantify compaction quality aspects. The particle movement (i.e., acceleration and Euler angles) was monitored by an innovative smart, wireless sensor resembling irregular particle shape during the compaction process. The relation between meso-scale particle movement and macro-scale compaction characteristics was established from laboratory test results. The methodology of the tests, experimental setup, materials, and data acquisition are all described in Section 2 of the paper. The analysis of the testing results is discussed in Section 3, followed by the summary and conclusions of the testing program.

## 2. Experimental Materials, Equipment, and Testing Program

### 2.1. Materials

**Figure 1:**
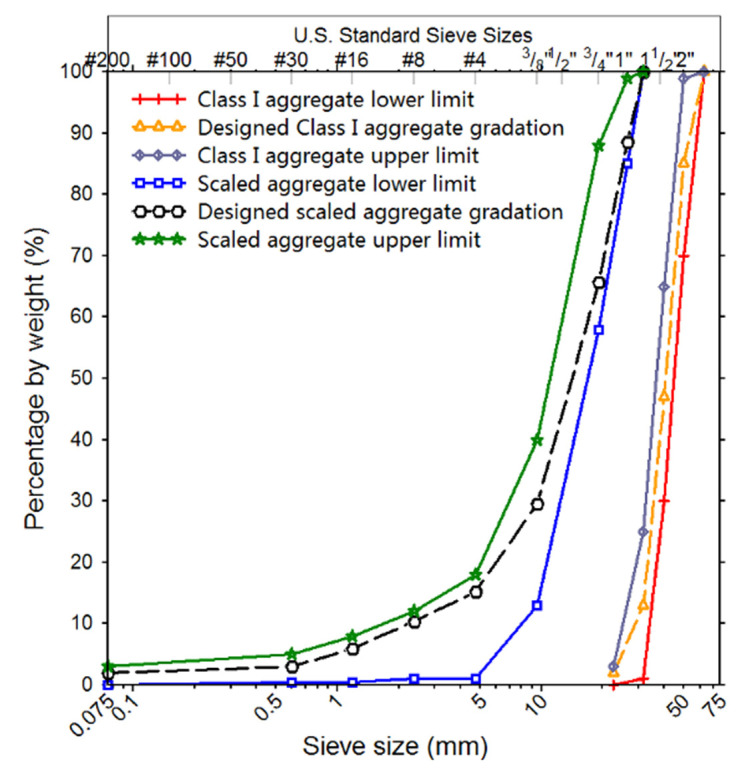
Illustration of original and scaled aggregate gradation curves.

The unbound aggregate materials tested in the study were collected from a commercial quarry located in Hunan province, China, and were rated as Class I crushed granite according to Chinese standards [30]. Figure 1 shows both original and scaled aggregate gradation curves used in this study, along with original and scaled gradation bands of Class I aggregates specified in the Chinese design code. The nominal maximum particle size of the original aggregate gradation is 63 mm, too large to be used in the commercial gyratory compactor available in this study, as the diameter of the mold of the gyratory compactor is 100 or 150 mm. To eliminate the size effect on aggregate compaction behavior, the maximum particle size should be at least five times smaller than the diameter of the gyratory specimen [31,32,33]. Therefore, the original aggregate gradation was scaled using the parallel similitude method adopted by Cui et al. [34,35]. The scaled gradations by this procedure were reported to well represent original gradations in terms of mechanical behavior [36,37,38]. Note that it is out of the scope of this study to verify whether or not the gradation scaling introduces a difference in compaction behavior by performing related laboratory element and model tests. Based on the gradation scaling procedure described, the scaled aggregate gradations were determined from the original ones. As described in Equation (1), the percentage of materials by weight passing through a specific grain size $D^{m}$ for scaled aggregate gradation has the same value as that corresponding to $D^{b}$ for original aggregate gradation.

(1) $$\frac{D^{b}-D_{min}^{b}}{D^{m}-D_{min}^{m}}=\frac{D_{max}^{b}-D_{min}^{b}}{D_{max}^{m}-D_{min}^{m}}=A$$

where $D_{max}$, $D_{min}$, and D are the maximum grain size, the minimum grain size, and the specific grain size, respectively; superscripts b and m denote original and scaled ballast gradations, respectively; and A is a constant. Based on the maximum and minimum grain sizes of original and scaled aggregate gradations, the constant A was calculated using Equation (2).

(2) $$A=\frac{D_{max}^{b}-D_{min}^{b}}{D_{max}^{m}-D_{min}^{m}}=\frac{63-22.4}{31.5-0.075}=1.29$$

### 2.2. Orthogonal Array Test Design

**Figure 2:**
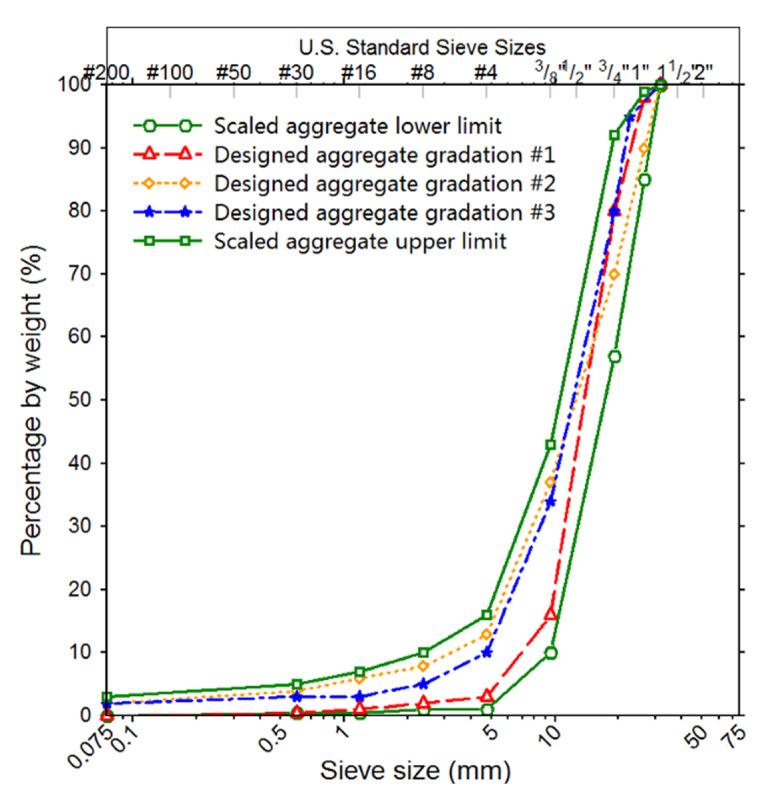
Three different types of scaled aggregate gradation curves studied.

The orthogonal design theory is an effective method to arrange multi-factor and multi-level experiments. Its advantage in laboratory testing is to minimize the number of cases in the entire test matrix, yet still maximize the test coverage and accuracy. In order to explore the optimal parameter combination of laboratory gyratory specimens of unbound permeable aggregates (i.e., moisture content, vertical pressure, and gradation), the orthogonal array test design was adopted. A series of conventional impact compaction tests were conducted in the laboratory to obtain the optimal moisture content of UPAB materials with scaled gradations of 1–3% [39]. Previous studies revealed that the vertical stress level observed during field compaction and in-service periods is from 100 to 700 kPa [21,40,41,42]. The details of the orthogonal array testing along with the test design scheme are shown in Table 1, which was obtained according to the principle of mixed-level orthogonal design. Note that the combinations of test parameters obtained from orthogonal array design are rational, but not unique. Specifically, the symbol A in Table 1 denotes the moisture content that has six different levels coded by numbers 1 through 6 of 1.2, 1.5, 2.0, 2.5, 3.0, and 3.5%, respectively; the symbol B denotes the vertical pressure that has three different levels coded by numbers 1 through 3 of 400, 600, and 800 kPa, respectively; and the symbol C denotes gradation that has three different types, as shown in Figure 2.

### 2.3. Gyratory Compaction

**Figure 3:**
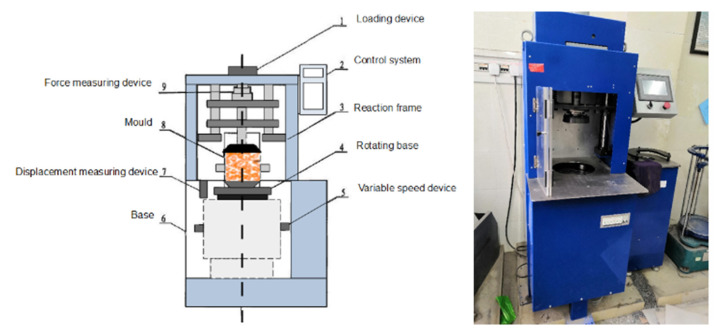
Illustration of different components of the gyratory compactor used.

The gyratory compactor adopted in this study is commonly used for compacting hot mix asphalt, as illustrated in Figure 3. It has the following main technical specifications: calibrated internal angle of 1.16° and rotational speed of 30 rpm. The diameter of the specimen is 150 mm. The real-time height data of gyratory specimens were recorded directly by the built-in data acquisition system of the gyratory compactor. Despite that there are currently no standardized test procedures for compacting soil with a gyratory compactor, four major variables are regarded influential for gyratory compaction quality, i.e., gyration angle, gyrations, vertical pressure, and gyration rate. Previous SHRP work by Cominsky et al. [43] indicated that little variation was obtained through different rates of rotation and this seemed to be applicable at any angle. The gyratory angle was previously found to affect soil compaction, and the void ratio of soil specimens became more stable when the angle was between 1.0 and 2.0 degrees [43]. Therefore, in this study, the gyratory angle and gyration rate were fixed at 1.16° and 30 rpm for all the specimens tested, respectively, while three different levels of vertical pressure were adopted. The complete record of specimen height and dry density change with the number of gyrations was obtained for each specimen during the gyratory compaction process.

### 2.4. Smart Sensors for Monitoring Particle Movement

**Figure 4:**
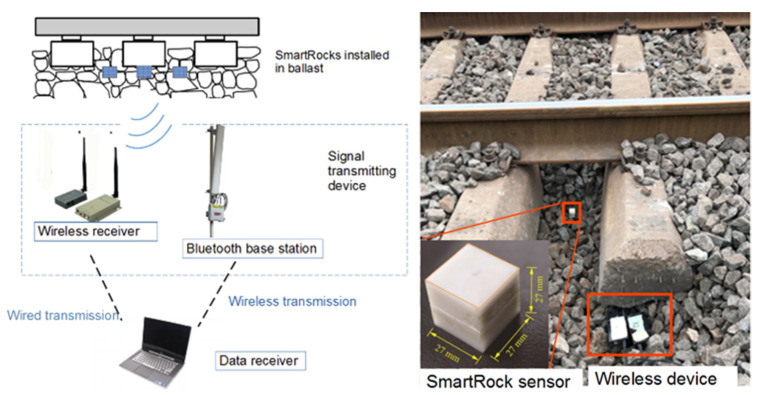
Illustration of the SmartRock (SR) sensing system (**left**) used in railroad track applications (**right**).

**Figure 5:**
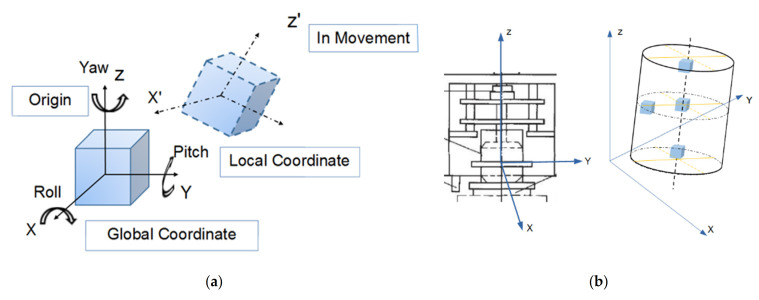
Illustration of (**a**) the two coordinate systems built in SR sensors and (**b**) placement locations of SR sensors within gyratory specimens.

Understanding meso-scale particle movement patterns under different compaction conditions could potentially provide insights into compaction mechanisms and technical guidance for field compaction and quality control. In this study, a new type of smart, wireless, and self-powered sensor with a 3D-printed shell resembling irregular shape of real aggregate particles, named as “SmartRock” sensor (abbreviated as SR sensor hereinafter), was adopted for real-time monitoring of meso-scale particle movement characteristics during gyratory compaction. Each SR sensor is powered by a built-in lithium manganese battery compatible with typical field in-service conditions in pavement and railroad applications. The capacity of the battery can support non-stop and continuous monitoring service for up to one day; however, such a non-stop monitoring need is extremely rare in reality, especially in pavement and railroad applications where traffic loads are repeated in nature. To extend the life of the battery, each SR sensor can be remotely controlled to hibernate once data collection is completed and to wake up once data collection is resumed. The discharged battery is replaceable and the other sensing components in SR sensors are reusable. Figure 4 shows the SR sensing system and its field applications in a ballasted railway track [25]. The major parameters that could be monitored by such SR sensors in real-time fashion include standard time, temperature, internal normal stress, 3D Euler angle, shear strain, and high-precision 3D acceleration, among others. Those data can be transmitted to either receivers or remotely-controlled roadside signal collectors by means of low-power Bluetooth or other communication protocols. The SR sensors contain two built-in coordinate systems, i.e., the global and local coordinate systems, as shown in Figure 5a [26]. In order to analyze particle movement at different locations of the gyratory specimen, four SR sensors were placed at designated positions illustrated in Figure 5b, i.e., upper center, middle center, lower center, and center side.

### 2.5. Particle Shape Quantification

**Figure 6:**
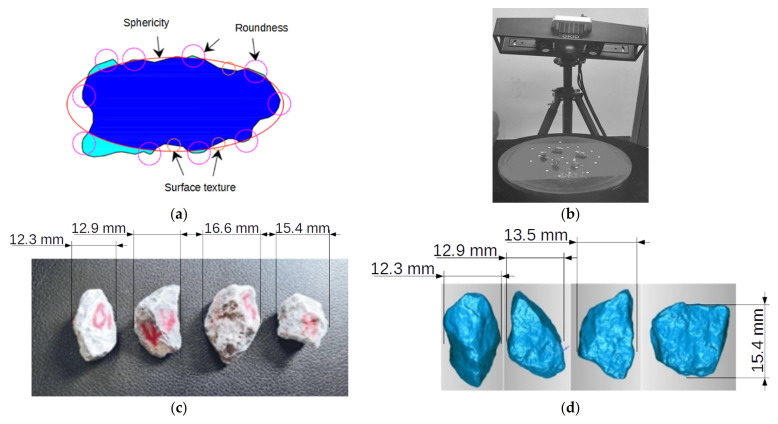
Illustration of particle morphology quantification: (**a**) 3D morphology definition; (**b**) 3D laser scanner used; (**c**) examples of real coarse particles; and (**d**) reconstructed 3D models of example particles.

Aggregate morphology including 3D form (or sphericity), angularity (or roundness), and surface texture (or roughness) characteristics (see Figure 6a) has long been recognized as influential for compaction and mechanical properties. The 3D laser scanner shown in Figure 6b was used to collect, analyze, and reconstruct the 3D geometries of individual coarse aggregate particles, from which particle morphology can be further quantified from reconstructed digital geometry [44,45]. As previously mentioned, the perimeter sphericity and convexity parameters were defined by Zheng [45] in Equations (3) and (4), respectively.

(3) $$S_{P}=\frac{P_{c}}{P_{s}}$$

(4) $$C_{x}=\frac{V_{i}}{V_{x}}$$

where *P_c_* denotes the perimeter of the circle having the same projected area as the particle, *P_s_* denotes the perimeter of the particle, *V_x_* denotes the volume of the convex hull of the particle, and *V_i_* denotes the volume of the largest inscribed sphere of the particle. To demonstrate and quantify the variation of particle morphology during gyratory compaction, a selection of coarse particles (retained on No. 4 or 4.75 mm sieve) were sampled from gyratory specimens prepared with scaled gradations. Then, those selected coarse particles (of which examples are shown in Figure 6c) were all digitalized for 3D shape (see Figure 6d) by the aforementioned laser scanner both before and after gyratory compaction.

**Figure 7:**
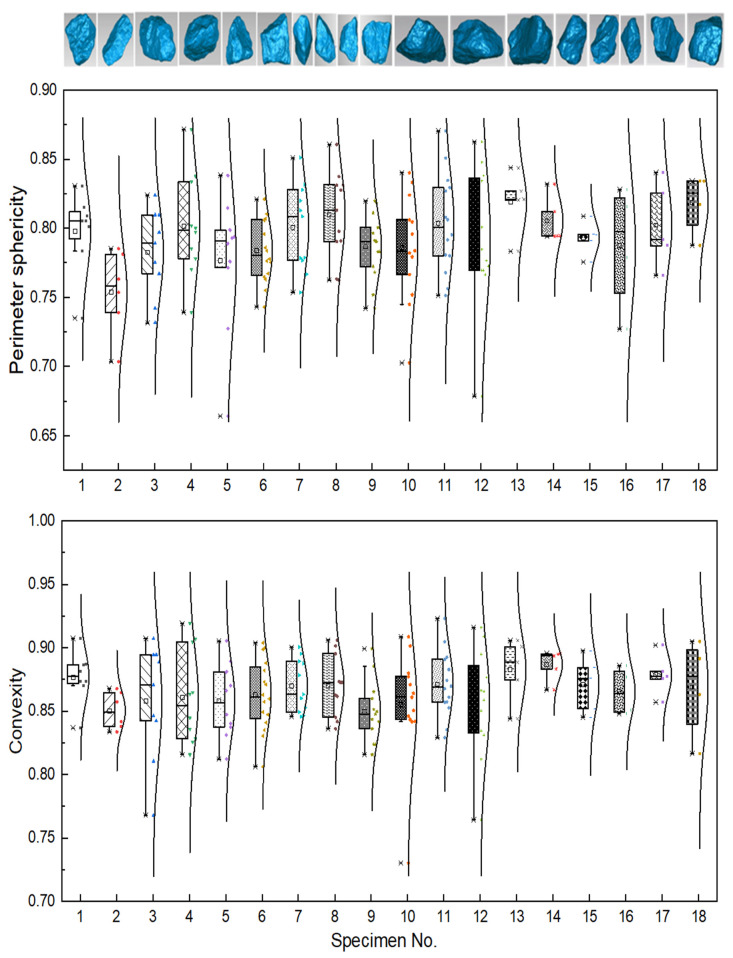
The visual and quantitative comparisons of particle perimeter sphericity and convexity values for all the gyratory specimens tested.

In this study, in order to minimize the effect of shape variation of coarse particles on compaction behavior, and thus reduce the potential total number of gyratory specimens required (if particle shape were introduced as an additional variable), the shape parameters of coarse aggregate particles of all the gyratory specimens were properly controlled at a similar level. As evident from Figure 7, the average values of perimeter sphericity of the selected particles from the 18 specimens range from 0.76 to 0.81, indicating that the perimeter sphericity of the particles of the 18 specimens is quite similar. The average convexity values of selected particles from the 18 specimens range from 0.85 to 0.88. It is quite obvious that the difference in convexity values of the 18 specimens is smaller than that in perimeter sphericity values. Therefore, the particles sampled from the 18 specimens possess similar geometry, and the effect of morphology variation of coarse particles on compaction behavior can be ignored.

## 3. Results and Analysis

### 3.1. The Determination of the Optimal Combination of Gyratory Compaction Parameters

**Figure 8:**
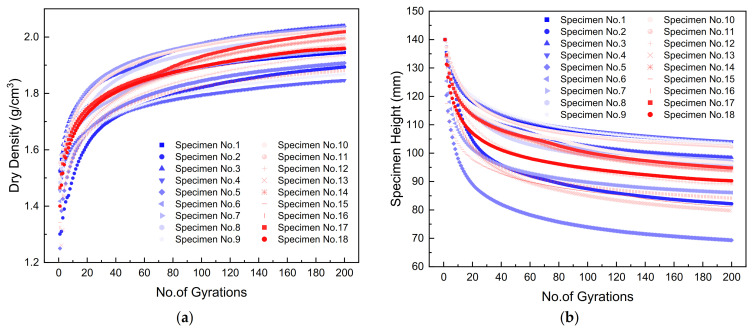
Specimen height and dry density changes versus the number of gyrations for scaled aggregate gradations at different levels of moisture content: (**a**) specimen height and (**b**) dry density.

**Figure 9:**
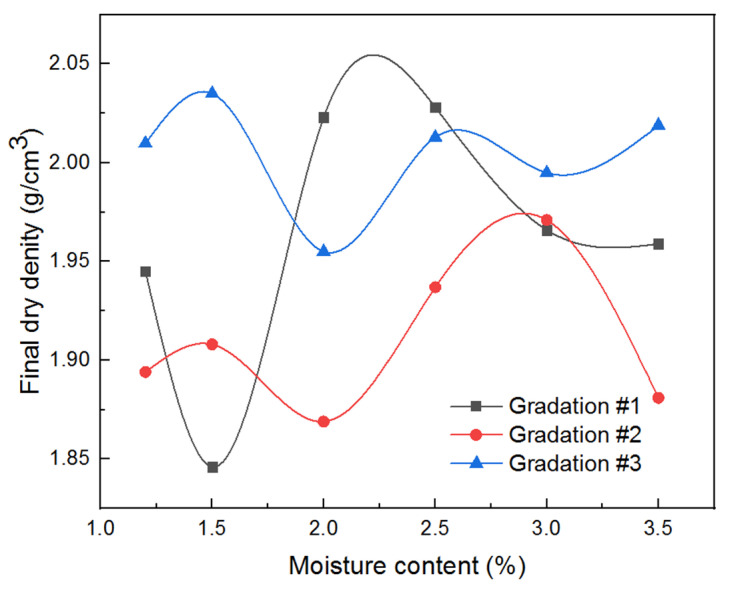
Moisture–density curves obtained from gyratory compaction tests for three different gradations.

The gyratory specimens were compacted by referencing the existing specification for hot mix asphalt, and the specimen height was recorded to calculate the achieved dry density. The gyratory compactor is equipped with LVDTs to measure the real-time vertical displacement (or equivalently height change) of the specimen. Prior to the gyratory compaction, the dry mass and moisture content of the specimen were recorded and known. The real-time dry density of the specimen during the gyratory compaction can then be calculated from the dry mass, the diameter, and the real-time height of the gyratory specimen. The real-time specimen height and dry density were plotted against gyrations in Figure 8. It can be observed from Figure 8 that, as the number of gyrations increases, the specimen height decreases continuously, with the decreasing rate reducing gradually; on the other hand, the real-time dry density value of the specimen increases continuously with an increasing number of gyrations, which indicates the gradual densification of the specimens achieved during the gyratory compaction process. The final achieved dry density values of gyratory specimens in the orthogonal array testing matrix were used as one of the primary compaction quality indices and are summarized in Table 2. The corresponding moisture–density curves of gyratory compaction were plotted in Figure 9 accordingly.

From Table 2 and Figure 9, it can be seen that the greatest final dry density value was achieved for the scaled aggregate gradation #3 with a moisture content of 1.5% under a vertical pressure of 800 kPa. It can be seen from Figure 9 that the compaction curves of the three different types of scaled aggregate gradations are different from that of the conventional fine-grained soils, i.e., the former exhibits multiple peaks instead of one single peak shown by the latter. This is probably attributable to the fact that the coarse-grained aggregate particles are more likely to break during the compaction process, thus resulting in staged, progressive structure changes of the aggregate specimens. The optimal moisture content values of the three gradations were different, i.e., 2.5, 3.0, and 1.5% for gradations #1, #2, and #3, respectively. This indicates that gradation could affect the optimal moisture content and maximum dry density of unbound permeable aggregates. The evaluation range R is an important index in the orthogonal array test. The larger the R value, the more important the corresponding factor. The specific definition is as follows:

(5) $$R=max\left\{m_{1},m_{2},m_{3},m_{4},m_{5},m_{6}\right\}-min\left\{m_{1},m_{2},m_{3},m_{4},m_{5},m_{6}\right\}$$

where *M_i_* is the sum of corresponding test results with the level number being *i* in any column; *s* is the number of occurrences of the *i*-th level in any column; and $m_{i}=M_{i}/s$.

From the values of range *R*, it can be seen that gradation has the greatest influence on gyratory compaction, followed sequentially by vertical pressure and moisture content. At the same time, variance analysis was performed by SPSS analysis software and the result is shown in Table 3. Comparing the significance of the three different factors, it was found that the gradation has the greatest influence and the moisture content has the least influence on the dry density of the aggregate materials with scaled gradations. The result is consistent with that from the statistical range analysis.

### 3.2. Distinct Stages of Gyratory Compaction

**Figure 10:**
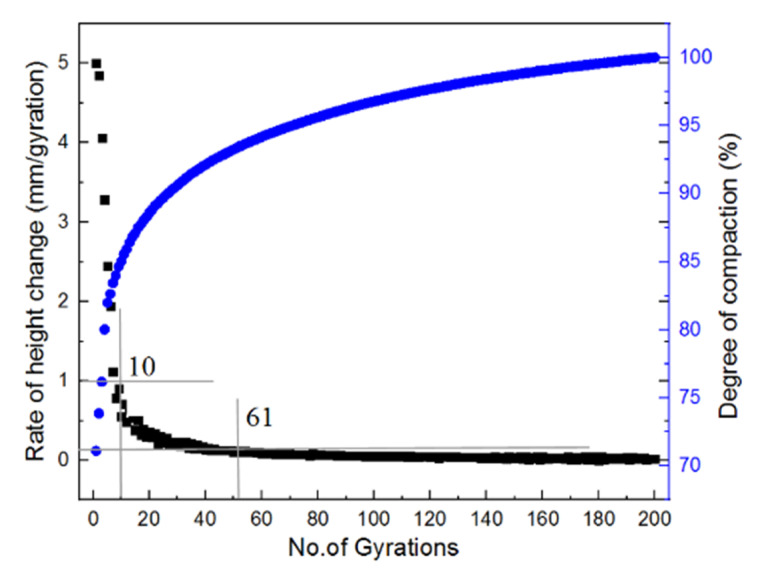
The rate of height change and degree of compaction versus the number of gyrations for the specimen with an optimal combination of gyratory compaction parameters.

**Figure 11:**
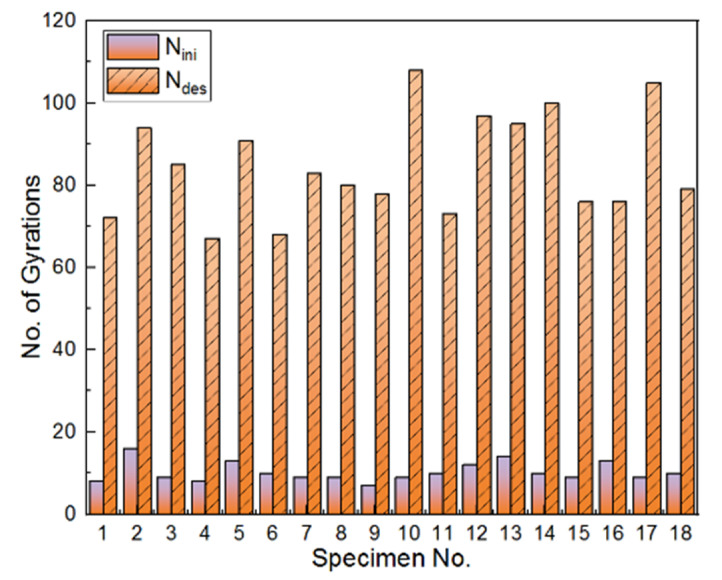
The histograms of the initial number (Nini) and design number (Ndes) of gyrations identified for different specimens in the orthogonal array matrix.

Zhang et al. [46] divided the entire compaction process into three stages by three threshold points corresponding to the initial number (*N_ini_*), the design number (*N_des_*), and the final number of gyrations, respectively. The rate of height change greater than 1 mm per gyration can be regarded as the first stage, 0.1 to 1 mm per gyration as the second stage, and 0 to 0.1 mm per gyration as the final stable stage. The second stage ranges between the initial number and the design number of gyrations, whereas the final stage ranges between the design number and the final number of gyrations. To take an example, the rate of height change and the degree of compaction of the specimen compacted with the optimal combination of gyratory parameters were calculated and plotted against the number of gyrations in Figure 10. It can be seen that the initial self-compaction stage is from 0 to 10 gyrations, the intermediate gyratory compaction stage is from 10 to 61 gyrations, and the final stable stage is from 61 to 200 gyrations. The two threshold points (*N_ini_* and *N_des_)* for all the specimens in the orthogonal array matrix are shown in Figure 11. The maximum difference of *N_in_i* values of 18 specimens is 12, whereas the maximum difference of *N_des_* values is 46. Therefore, it can be inferred that the *N_ini_* values are relatively close among different specimens; however, the *N_des_* values show somewhat considerable variations. This infers that physical properties and compaction parameters of unbound permeable aggregates significantly affect the second stage of gyratory compaction and corresponding compaction quality.

### 3.3. Characteristics of Gyratory Compaction

The gyratory compaction process can be divided into three distinct stages according to the above-mentioned analysis. The first stage or stage I ($N_{sta}-N_{ini}$) resulted from abrasion at the aggregate particle contacts and is analogous to the post-construction stage. The second stage (or stage II) is featured by the rearrangement of the particle grain ($N_{ini}-N_{des}$), the core stage of the compaction process. The third stage (or stage III) is regarded as the stable or final compaction stage where insignificant improvement in compaction quality (e.g., the degree of compaction) and increasing fracture of particle grains are observed as the number of gyrations further increases. It should be noted that, during the second and third stages, gravity-induced self-compaction still exists, but it does not play a leading role. Hence, stage II is mainly studied herein as compared with stages I and III.

**Figure 12:**
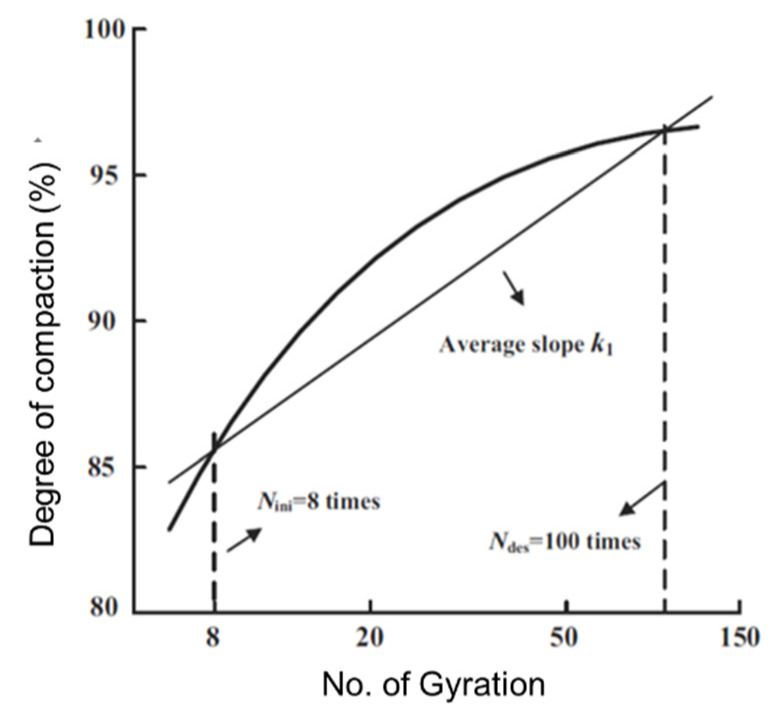
Illustration of the definition of average slope *K*_1_ from the compaction curve of the degree of compaction versus the number of gyrations [46].

As illustrated in Figure 12, the curve of the degree of compaction versus the number of gyrations, which is displayed on a semi-logarithmic plot, can be used to define the average slope *K*_1_ using Equation (6) [46]. The parameter *K*_1_ can then be used to assess the compaction state, with a higher *K*_1_ value indicating better compaction quality.

(6) $$K_{1}=\frac{\%\gamma_{d}@N_{des}-\%\gamma_{d}@N_{ini}}{lnN_{des}-lnN_{ini}}$$

where $\%\gamma_{d}@N_{des}$ and $\%\gamma_{d}@N_{ini}$ are the degree of compaction achieved at the design number and initial number of gyrations, respectively.

To take as an example the gyratory specimen with the scaled aggregate gradation #3 under 800 kPa vertical pressure, the threshold points defining the three compaction stages are the 10th, 61st, and 200th gyration, respectively. Therefore, the *K*_1_ value is calculated from Equation (7) as follows.

(7) $$K_{1}=\frac{94.85-84.85}{ln61-ln10}=5.53$$

**Figure 13:**
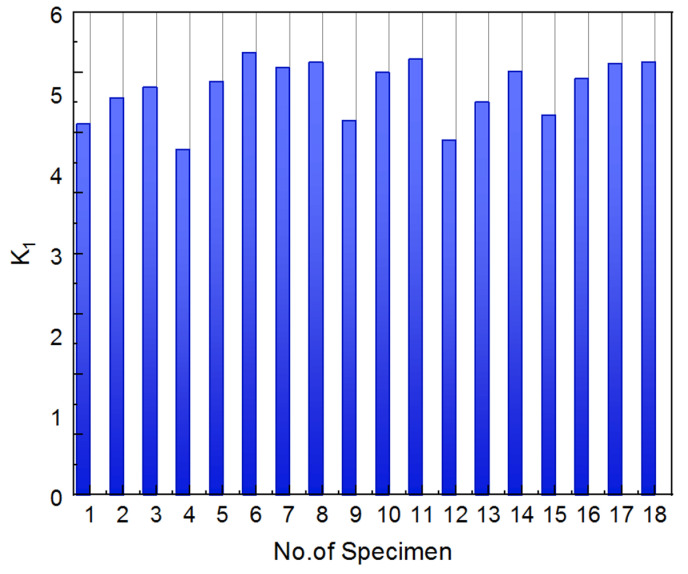
The calculated *K*_1_ values of 18 different specimens.

Figure 13 illustrates the calculated *K*_1_ value for 18 different specimens. It can be seen from Figure 13 that the *K*_1_ value of the specimen No. 6 with the optimal combination of gyratory parameters is relatively greater than those of other different specimens. This actually proves the rationality of the orthogonal array test design and the reliability of the obtained optimal combination of gyratory parameters.

## 4. The Characteristics of Meso-Scale Particle Movement

The movement data of individual coarse particles (i.e., acceleration and Euler angle) monitored by the SR sensors were linked to compaction behavior of aggregate materials with scaled gradations during the gyratory compaction with a goal to reveal the underlying mechanism of gyratory compaction of unbound permeable aggregates.

### 4.1. Acceleration of Particles at Different Locations

**Figure 14:**
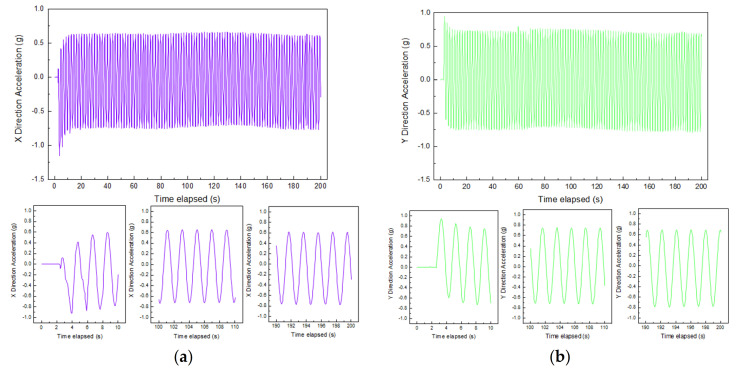

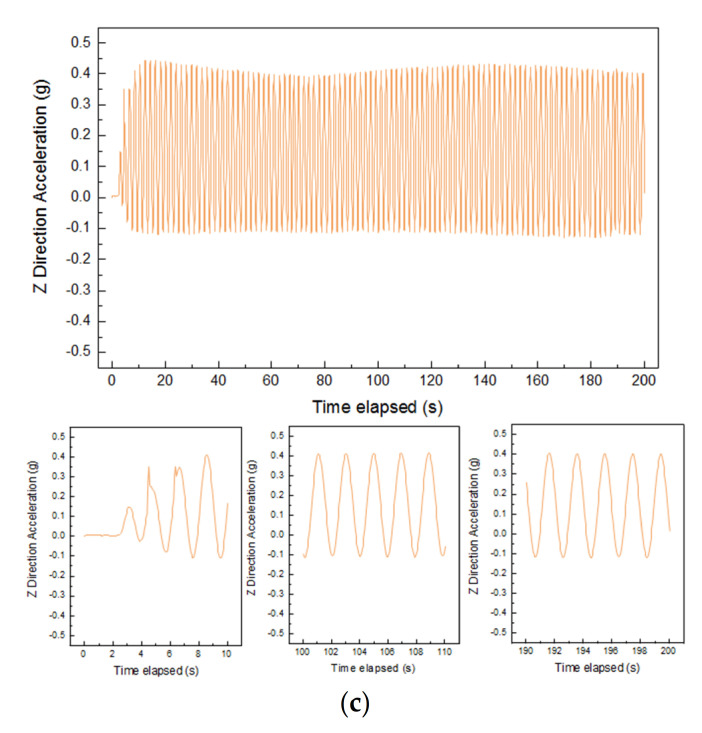
Acceleration results measured in three different directions of at the middle center of the specimen with optimal combination of gyratory parameters: (**a**) X direction, (**b**) Y direction, and (**c**) Z direction.

The acceleration data recorded by the SR sensors placed in the middle center of the specimen with optimal combination of gyratory parameters were analyzed to compare and evaluate particle acceleration response at different stages of gyratory compaction. The raw signals were first subjected to the start-from-zero processing and the low-pass filtering. The time history curves of accelerations in three directions of X, Y, and Z are plotted in Figure 14, including the overall time history curves of the first 200 s gyration and the selected local time history curves from the first stage (represented by 0–10 s), the second stage (represented by 100–110 s), and the third stage (represented by 190–200 s). Note that the acceleration values in the Z direction are net values with the gravitational acceleration (g) deducted, and that the negative sign denotes the direction of acceleration opposite to the positive direction of the corresponding axis shown in Figure 14. It can be seen that the overall time history curve exhibits 100 peaks and troughs within the duration of 200 s. This indicates that particle acceleration has a clear periodic pattern that matches the period of 2 s (or equivalently the frequency of 0.5 Hz) of the gyratory compactor (of which the gyration speed is 30 rpm). Hence, the parametric settings of the gyratory compactor could potentially affect meso-scale particle movement patterns.

The particle accelerations in the X, Y, and Z directions all increase rapidly from zero during the first stage of gyratory compaction (i.e., self-compacting stage). The accelerations in the X and Y directions increase faster than the Z direction and gradually shift from single-amplitude vibration to dominant double-amplitude vibration (with zero value being the mean position), while the acceleration in the Z direction remains to be dominant single-amplitude vibration. This indicates that the translational movement of particles in the X and Y directions is manifested as the lateral self-compaction of internal pores within the gyratory specimen, while the translational movement of particles in the Z direction is dominated by vertical compression of internal pores. During the second stage (i.e., the gyratory compaction stage), the acceleration variations in the X, Y, and Z directions tend to be stable, and the acceleration in the lateral direction (i.e., the X or Y direction) is significantly greater than that in the vertical direction (i.e., the Z direction). This is because particles are subjected to obvious shearing action by gyratory force and the effect of such shearing force on particle acceleration is greater than the vertical pressure. The particle accelerations in the three directions during the third stage (i.e., the stable compaction stage) are close to those during the second stage and are mainly affected by the compaction parameters of the gyratory compactor, and the internal structure of the gyratory specimen has become quite stable.

### 4.2. Characteristic Acceleration Value of Particles at Different Locations

**Figure 15:**
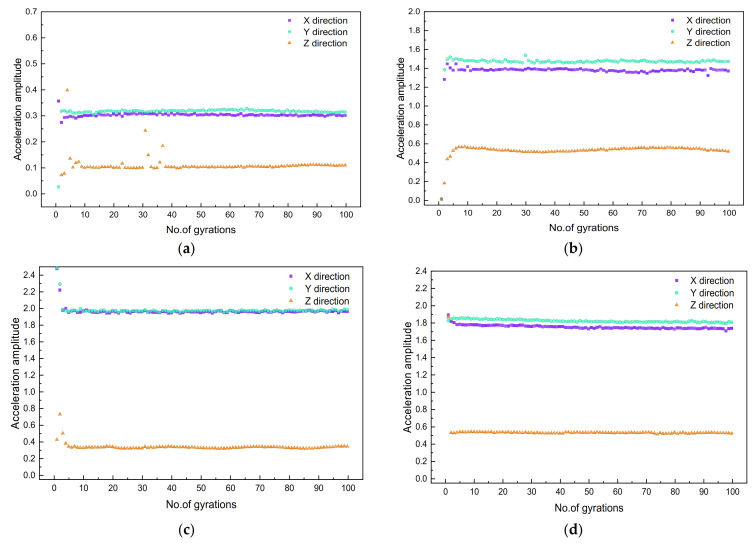
Acceleration characteristic value results measured at four different positions of the specimen with optimal combination of gyratory parameters: (**a**) lower center, (**b**) middle center, (**c**) upper center, and (**d**) center side.

The difference between the peaks and troughs of the time history curve of particle acceleration within each period of interest is taken as the characteristic acceleration value to compare the differences of particle movement at different positions of the gyratory specimen. Such characteristic particle acceleration values at four different positions are plotted against the number of gyrations in Figure 15. It can be seen that the characteristic acceleration values in the X and Y directions are significantly greater than those in the Z direction, indicating that the shearing action by gyratory force plays a more important role than the vertical pressure during the process of gyratory compaction. To be specific, the following observations can be further made: first, the characteristic acceleration values in the Z direction at the lower center and middle center positions of the gyratory specimen increase rapidly with the increasing number of gyrations during the initial stage, which is consistent with the dry density change of the specimen, indicating that the rapid movement of the middle and lower particles promotes a rapid increase in dry density of the specimen; second, the particle acceleration in the Z direction at the upper center position of the specimen shows a rapid decrease, mainly because particles in the upper center position are close to the load plate. The rate of the change in specimen height decreases gradually with the number of gyrations, indicating that particle acceleration at this position is mainly affected by the rate of the decrease in specimen height; and third, the particle acceleration in the Z direction at the center side position, as compared with those at the center positions, appears not to be affected by the change in specimen height and remains stable during the process of gyratory compaction.

The particle accelerations in the X and Y directions exhibit a weak correlation with the decrease in specimen height as compared with that in the Z direction, because the former is mainly affected by the shearing action of gyratory force. The fluctuation of particle accelerations in the X and Y directions during the initial compaction stage is greater than that during the other two stages, further indicating that the internal pores among particles during the initial compaction stage are larger. The particles need to be adjusted laterally to make the pore distribution more uniform in addition to vertical compression. By comparing the characteristic acceleration values of particles at three different center positions of the specimen, it can be seen that the greatest acceleration occurs at the upper center position, followed sequentially by the middle center and lower center positions. This demonstrates that particle acceleration shows a decreasing trend from top to bottom of the specimen. The characteristic acceleration values in the X and Y directions at the side position are greater than those at the center position, because the side position is farther from the main gyratory shaft and the shearing effect at this position is more pronounced during gyratory compaction. For the sake of brevity, only the meso-scale particle movement results recorded by the SR sensors placed at four different locations of the specimen with optimal combination of gyratory parameters are plotted to illustrate their linkage with macro-scale compaction characteristics, while the results of other gyratory specimens in the orthogonal array matrix are skipped herein, but indeed revealed similar trends, as described subsequently.

The thresholding number of gyrations (A.K.A. the staged point) where the characteristic acceleration values in the Z direction start to become stable was taken as the staged point, and the staged points of particle accelerations at four different positions of the total eighteen specimens were counted. As listed in Table 4, except for a few specimens, little difference is found among the staged points corresponding to the same position of the majority of the eighteen specimens. Therefore, the average staged point (i.e., average number of gyrations) of particle accelerations at different positions was obtained by averaging the staged points of different specimens. The average staged point of particle accelerations at four different positions ascends in the order of the upper center, middle center, lower center, and center side, which indicates that the gyratory compaction action propagates gradually from upper center to lower center and from the center to the side of the gyratory specimen.

### 4.3. Relationship between Particle Acceleration and Rate of Change in Specimen Height

**Figure 16:**
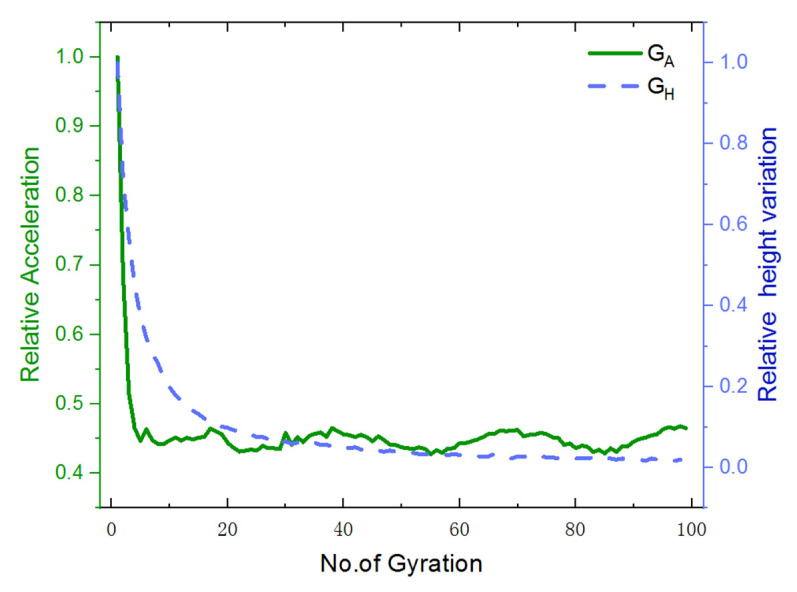
The relative specimen height %*H* and the relative particle acceleration *%A* plotted against the number of gyrations.

In order to further analyze the relationship between particle acceleration and compaction characteristics, the relative height variation %*H* and the relative acceleration %*A*, which are calculated from Equation (8) and Equation (9), respectively, are plotted against the number of gyrations in Figure 16. It can be seen that both %*H* and %*A* curves descend drastically during the initial cycles of gyratory compaction and then gradually become stable.

(8) $$\%H=\frac{V_{H}}{V_{Hmax}}\;\times \;100\%$$

(9) $$\%A=\frac{A}{A_{max}}\;\times \;100\%$$

where $V_{H}$ is the specimen height variation during each cycle of gyration, $V_{Hmax}$ is the maximum specimen height variation, *A* is the characteristic acceleration value of the specimen during each cycle of gyration, and $A_{max}$ is the maximum characteristic acceleration value of the specimen.

The influences of gradation, moisture content, and vertical pressure on particle accelerations at different positions of the gyratory specimen were evaluated from the variance analysis of the orthogonal array test, of which the results are summarized in Table 5. It can be seen that the particle acceleration at the upper center position has the most significant correlation with vertical pressure, further indicating that the upper particles are closest to the load plate of the gyratory compactor. The particle acceleration at the middle center position has significant correlation with gradation, moisture content, and vertical pressure, thus indicating that particle acceleration at this position is affected by the combination of these three factors. The particle acceleration at the lower center position has more significant correlation with the gradation, indicating that the inter-particle packing structure of the specimen does not change significantly. It shows that the coarse particles at the lower center position are less prone to breakage than those at the upper center position, as evidenced from the comparison of the correlations between the accelerations at both upper and lower center positions and the gradation.

### 4.4. Euler Angle of Particles at Different Locations

**Figure 17:**
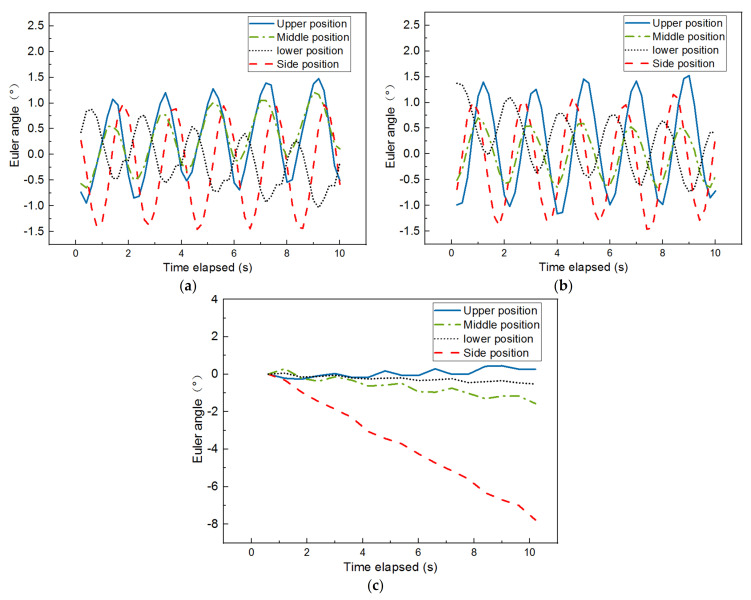
Euler angle results measured at four different positions of the specimen with optimal combination of gyratory parameters: (**a**) X direction, (**b**) Y direction, and (**c**) Z direction.

The Euler angle is the integration of three independent angles used to uniquely determine the position of a rigid object rotating about a fixed axis. It consists of roll, pitch, and yaw, as shown in Figure 5a. Figure 17 plots the time histories of the Euler angle in three dimensions recorded by the SR sensors placed at four different locations of the specimen with optimal combination of gyratory parameters. While the same periodic feature as previously described is found from the time histories of the Euler angles in both the X and Y directions, no such periodic pattern is observed in the Z direction. There seems to exist a phase lag between the Euler angle response at the lower position and those at the other three positions. This is expected as the rotational shear force is applied at the bottom plate of the specimen. The Euler angle response in the Z direction at the center side position keeps increasing, whereas such responses at the other three positions fluctuate around zero value. Hence, it appears that the Euler angle response in the Z direction at the center side position, which increases with the increasing densification level, could be used as a meso-scale indicator of gyratory compaction quality. The amplitudes of the periodic Euler angle responses in the X and Y directions at the upper center and center side positions are close and greater than those at the other two positions, which is consistent with the result of particle acceleration.

### 4.5. Relative Rotation of Particles at Different Locations

During the course of gyratory compaction, meso-scale particle movement mainly includes translation and rotation. The translation of particles is shown as the decrease in specimen height, while the rotation of particles reflects the inter-particle contact and interlocking. In this study, the rotation of particles is quantified and analyzed as the relative rotation. As expressed in Equation (10), the relative rotation in each of the three dimensions is defined as the difference between the maximum and minimum values of the Euler angle within each cycle. The results of such relative rotation values are plotted against gyration cycles in Figure 16. Note that the so-defined relative rotation measures maximum movement angle of particles within each cycle in X, Y, and Z directions, respectively.

(10) $$R=R_{Emax}-R_{Emin}$$

where *R* is the relative rotation and $R_{Emax}$ and $R_{Emin}$ are the maximum and minimum values of the Euler angle during each cycle, respectively.

**Figure 18:**
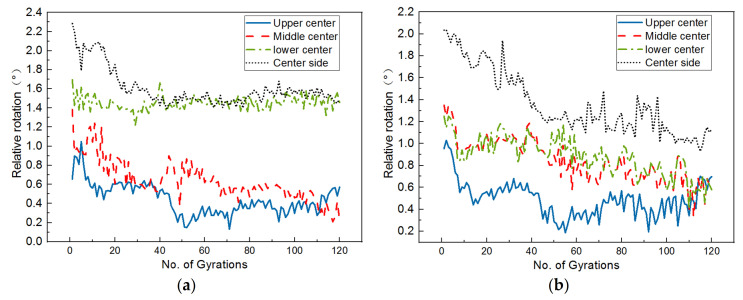

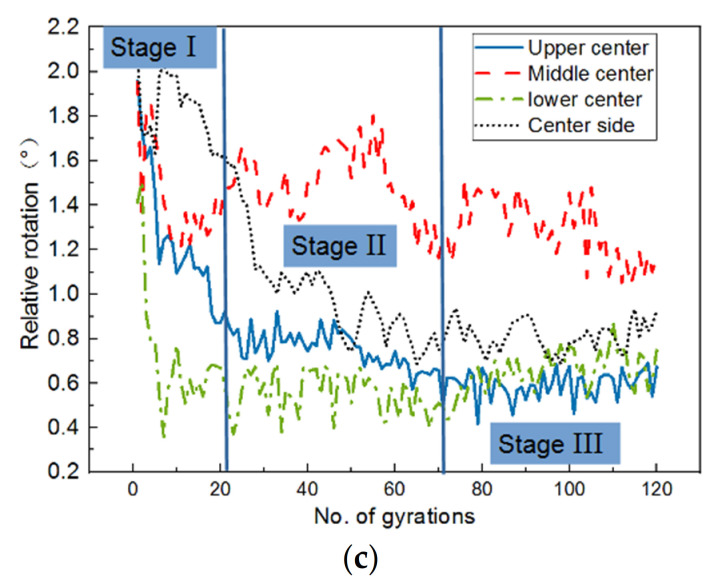
Relative rotation results measured at four different positions of the specimen with optimal combination of gyratory parameters: (**a**) X direction, (**b**) Y direction, and (**c**) Z direction.

From Figure 18, it can be seen that most of the curves of relative rotation versus gyration cycles exhibit a sharp reduction during the initial stage and then gradually become stable during the subsequent stage. Hence, such curves can be divided into the aforementioned three distinct stages, i.e., the self-compaction stage (or stage I), the transition stage (or stage II), and the stable stage (or stage III), as illustrated in Figure 18c. The three stages of particle rotation are similar to those of specimen height or achieved dry density. Obviously, smaller and more stable relative rotation values of coarse particles tend to indicate stronger inter-particle contact and interlocking, and thus less potential of further densification. This further supports the innovative and rational use of meso-scale particle movement to characterize the gyratory compaction process and control compaction quality. Note that the maximum relative rotation values recorded at those four different positions appear to occur in different directions, which suggests the position-dependent feature of particle rotation. The curves of relative rotation versus gyration cycles corresponding to the upper center and center side positions exhibit the three distinct stages much more clearly; therefore, the potential use of relative rotation response at these two positions as a meso-scale indicator of gyration compaction quality is supported.

### 4.6. Relationship between Relative Rotation and Dry Density Change

**Figure 19:**
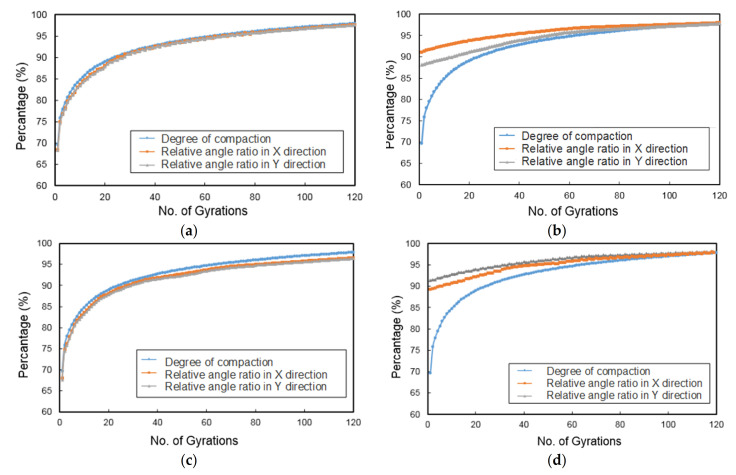
The relative rotation ratio and the degree of compaction at different positions of the specimen versus the number of gyrations: (**a**) upper center, (**b**) middle center, (**c**) lower center, and (**d**) center side.

Similar to the definition of the degree of compaction (i.e., the achieved dry density divided by the maximum dry density obtained under the same level of compaction energy), the relative rotation ratio is defined as the ratio of the relative rotation measured at the final (i.e., the 200th) gyration to that of the specific gyration of interest. Figure 19 shows the relative rotation ratio calculated at the four different positions of the specimen against the number of gyrations. As the number of gyrations increases, the relative rotation ratio increases with a gradually decreasing rate until reaching a relatively stable plateau. The increasing trend of the relative rotation ratio is generally consistent with that of the degree of compaction; particularly, the curves of the relative rotation ratio and the degree of compaction almost coincide with each other at the upper center and lower center positions. Therefore, the relative rotation ratio (especially at the upper center position) may be used as another meso-scale indicator of gyratory compaction quality.

### 4.7. Quantification of Particle Abrasion and Breakage

**Figure 20:**
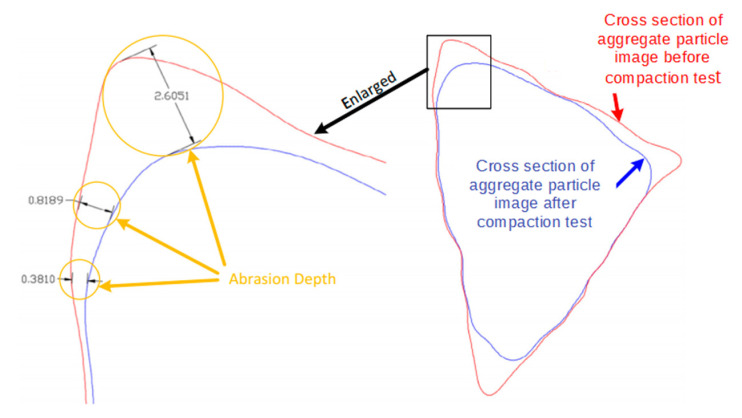
Illustration of abrasion depth calculation.

**Figure 21:**
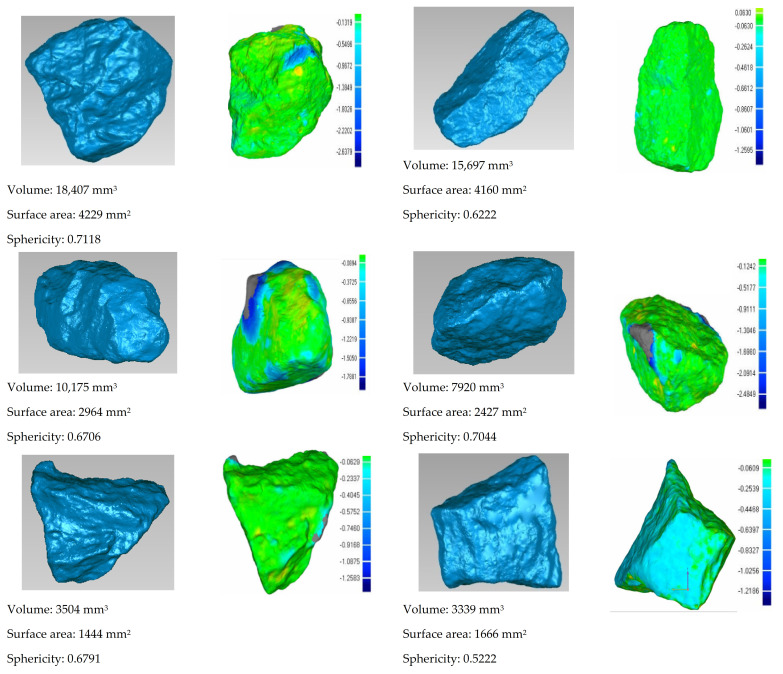
Contour plots of abrasion depth of example coarse particles determined from the comparison of digitized particle shape before and after gyratory compaction.

The particle abrasion and breakage are also some of the important aspects of compaction characteristics that deserve assessment and investigation, particularly for unbound permeable aggregates. Few studies have quantified particle abrasion and breakage during compaction, partly owing to the lack of proper techniques. With the advent of 3D laser scanning, it becomes possible to accurately digitize coarse particles with a 3D irregular shape. In this study, a selection of coarse particles retained on a No. 4 or 4.75 mm sieve were randomly picked, marked, and then digitized via the 3D laser scanner before and after gyratory compaction for each of the 18 specimens included in the orthogonal array testing matrix. The goal was to quantify particle shape variation caused by compaction. As shown in Figure 20, the abrasion depth of one point on the aggregate surface is defined and calculated as the distance between aggregate particle outlines before and after gyratory compaction that corresponds to this point. Among those different abrasion depth values, the maximum one is defined as the maximum abrasion depth to quantify the degree of abrasion experienced by each particle. The calculated abrasion depth values of selected aggregate particles were color-coded as contour plots and are shown in Figure 21. It can be seen from Figure 21 that the corners and ridges of such particles are more prone to abrasion, as indicated by the darker colors.

**Figure 22:**
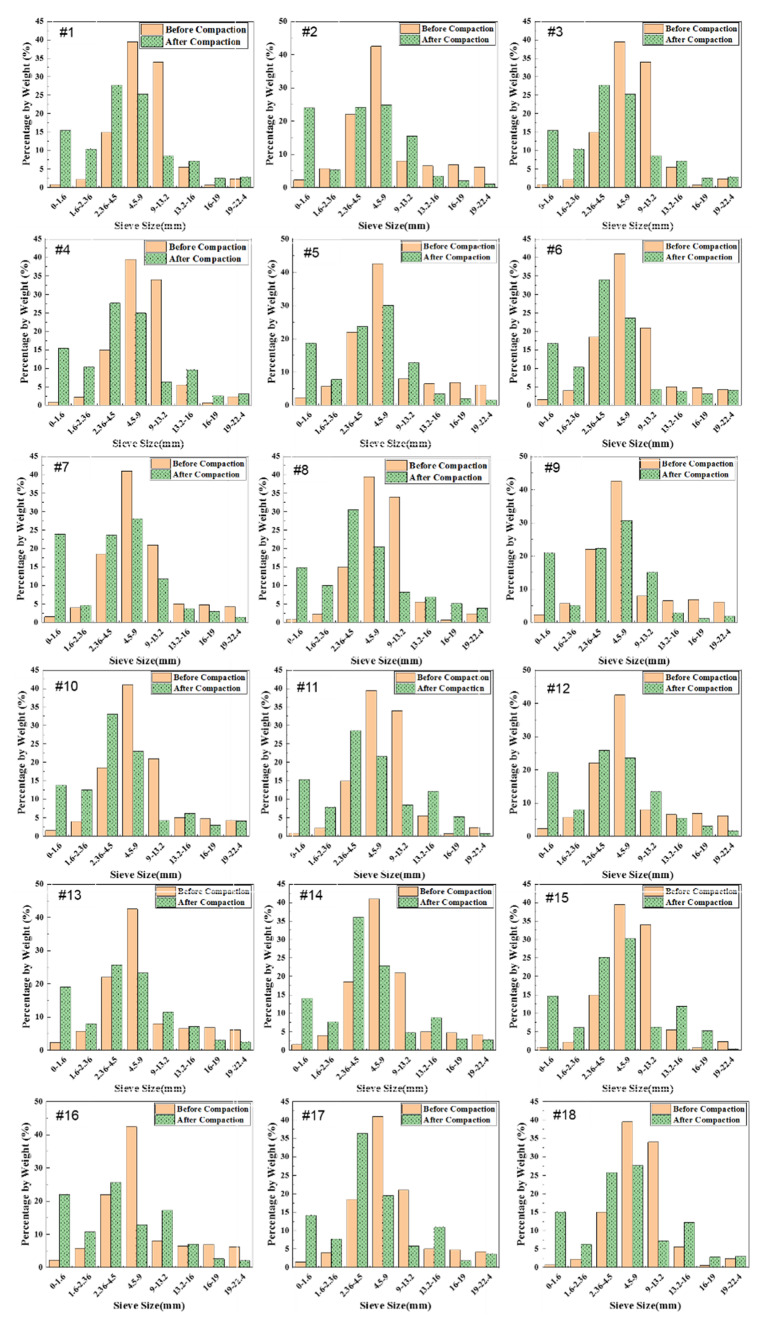
Histograms of particle size distributions before and after gyratory compaction for the eighteen specimens included in the orthogonal array testing matrix.

**Figure 23:**
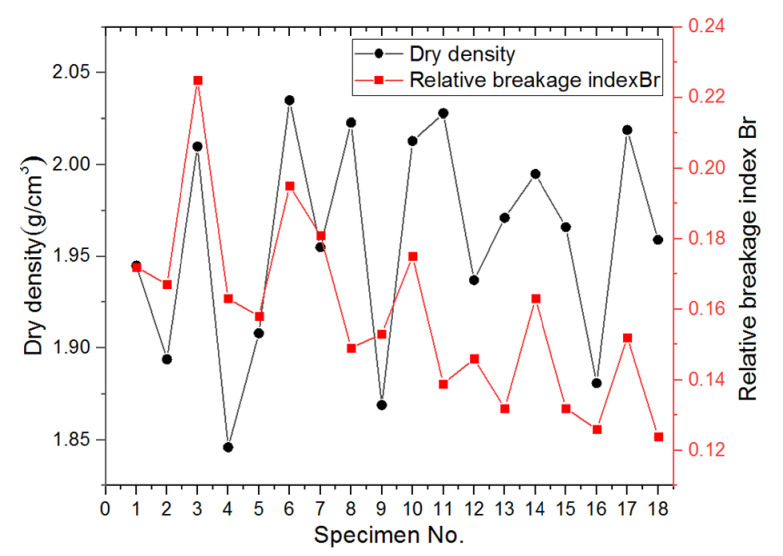
The relative breakage index Br versus final achieved dry density for 18 gyratory specimens included in the orthogonal array testing matrix.

Besides particle abrasion, particle breakage, another common phenomenon during compaction, was also quantified by performing mechanical sieve analysis on the aggregate materials of each gyratory specimen before and after compaction and comparing the gradation curves obtained. Figure 22 shows the histograms of particle size distributions before and after gyratory compaction for the 18 specimens included in the orthogonal array testing matrix. It seems that the greatest variation in particle size occurs approximately within the size range of 2.36 to 13.2 mm, thus indicating that particles within this size range are more susceptible to particle abrasion and/or breakage. The relative breakage index (Br) proposed by Hardin [47] was calculated for each of the 18 specimens from the difference of gradation curves before and after gyratory compaction. The coefficient of curvature (Cc) and coefficient of uniformity (Cu) values were also calculated for the gradation curves before and after gyratory compaction, respectively. It was found that the Cc values of the post-compaction gradation curves of the 18 specimens are all greater than 15 and the Cu values are greater than 40. Therefore, the post-compaction gradations of these 18 specimens are still regarded as poorly-graded. Figure 23 demonstrates the relationship between the final achieved dry density and the relative breakage index for all the 18 specimens. A rough consistency is observed between these two parameters, i.e., higher final achieved dry density approximately corresponds to greater relative breakage index. Hence, additional caution may need to be exerted when compacting the permeable unbound aggregates as studied herein in order to reach a cost-effective balance between compaction quality and particle breakage potential.

## 5. Discussion

Particle movement was obtained using the SmartRock sensors placed at different positions to analyze the gyratory compaction characteristics. According to the testing results, the gyratory compaction process can be divided into three different stages as follows:

- Stage I, self-compaction stage. During this stage, particles exhibit large relative rotation with a nearly linear decrease rate, resulting in significant height reduction (or density increase).
- Stage II, transition stage. During this stage, particle movement is restricted as represented by the reduced relative rotation. However, compaction still continues as represented by the stable acceleration, resulting in further reduction in specimen height.
- Stage III, stable stage. During this stage, particle movement is significantly restricted by the compaction-induced packing structure, and very minimal height (or density) change is further achieved.

Such particle rotation patterns and the division of compaction stages are consistent with the research findings reported by Liu et al. [29], who studied particle rotation characteristics of asphalt mixtures subjected to gyratory compaction. Meanwhile, their findings also indicated that the particles at the middle center position had a much higher degree of compaction than those at the lower center position, and thus reached the stable compaction stage slightly earlier than those located at the corner.

## 6. Summary and Conclusions

Laboratory and field compaction quality is assessed conventionally from physical indices such as dry density and void ratio (or porosity) only, which are still insufficient to disclose the compaction mechanisms. By designing a testing matrix from the orthogonal array theory, this paper explored the potential use of gyratory compaction as an effective method for compacting unbound permeable aggregate materials, and further attempted to link quality aspects of gyratory compaction with meso-scale particle movement monitored by an innovative wireless particulate sensing technology. Particle abrasion and breakage were also quantified via the 3D laser scanning technology. Based on the analysis results of laboratory compaction tests, the following major conclusions can be drawn:

- The best compaction performance was achieved for the gyratory specimen with the scaled aggregate gradation #3, a moisture content of 1.5%, and a vertical pressure of 800 kPa.
- Among the influential parameters studied, gradation was found to have the greatest influence on gyratory compaction quality, followed sequentially by vertical pressure and moisture content.
- The gyratory compaction process can be consistently divided into three distinct stages according to both macro-scale performance indicators and meso-scale particle movement characteristics.
- Meso-scale particle movement characteristics at the upper center of the specimen are promising indicators of compaction quality.
- Extreme caution is needed when compacting unbound permeable aggregates in order to reach a cost-effective balance between the quality of compaction and the extent of particle breakage.

Further work is currently underway to further validate the findings made in this study, explore the feasibility of using the SR sensors to monitor real-time density change during field compaction applications, and seek promising measures to improve both laboratory and field compaction quality of unbound permeable aggregate materials.

## Figures and Tables

**Table 1:** The orthogonal design scheme for laboratory gyratory compaction testing.

Specimen Number	Moisture Content (A, %)	Vertical Pressure (B, kPa)	Gradation Type (C)	Combination Code
1	1.2 (1)	400 (1)	#1 (1)	A_1_B_1_C_1_
2	1.2 (1)	600 (2)	#2 (2)	A_1_B_2_C_2_
3	1.2 (1)	800 (3)	#3 (3)	A_1_B_3_C_3_
4	1.5 (2)	400 (1)	#1 (1)	A_2_B_1_C_1_
5	1.5 (2)	600 (2)	#2 (2)	A_2_B_2_C_2_
6	1.5 (2)	800 (3)	#3 (3)	A_2_B_3_C_3_
7	2.0 (3)	600 (2)	#3 (3)	A_3_B_2_C_3_
8	2.0 (3)	800 (3)	#1 (1)	A_3_B_3_C_1_
9	2.0 (3)	400 (1)	#2 (2)	A_3_B_1_C_2_
10	2.5 (4)	600 (2)	#3 (3)	A_4_B_2_C_3_
11	2.5 (4)	800 (3)	#1 (1)	A_4_B_3_C_1_
12	2.5 (4)	400 (1)	#2 (2)	A_4_B_1_C_2_
13	3.0 (5)	800 (3)	#2 (2)	A_5_B_3_C_2_
14	3.0 (5)	400 (1)	#3 (3)	A_5_B_1_C_3_
15	3.0 (5)	600 (2)	#1 (1)	A_5_B_2_C_1_
16	3.5 (6)	800 (3)	#2 (2)	A_6_B_3_C_2_
17	3.5 (6)	400 (1)	#3 (3)	A_6_B_1_C_3_
18	3.5 (6)	600 (2)	#1 (1)	A_6_B_2_C_1_

**Table 2:** The final achieved dry density results from gyratory compaction tests with orthogonal array testing matrix.

Specimen Number	Moisture Content (A, %)	/	Vertical Pressure (B, kPa)	Gradation Type (C)	Final Achieved Dry Density (g/cm^3^)
1	1.2 (1)	1	400 (1)	#1 (1)	1.945
2	1.2 (1)	2	600 (2)	#2 (2)	1.894
3	1.2 (1)	3	800 (3)	#3 (3)	2.010
4	1.5 (2)	1	400 (1)	#1 (1)	1.846
5	1.5 (2)	2	600 (2)	#2 (2)	1.908
6	1.5 (2)	3	800 (3)	#3 (3)	2.035
7	2.0 (3)	1	600 (2)	#3 (3)	1.955
8	2.0 (3)	2	800 (3)	#1 (1)	2.023
9	2.0 (3)	3	400 (1)	#2 (2)	1.869
10	2.5 (4)	1	600 (2)	#3 (3)	2.013
11	2.5 (4)	2	800 (3)	#1 (1)	2.028
12	2.5 (4)	3	400 (1)	#2 (2)	1.937
13	3.0 (5)	1	800 (3)	#2 (2)	1.971
14	3.0 (5)	2	400 (1)	#3 (3)	1.995
15	3.0 (5)	3	600 (2)	#1 (1)	1.966
16	3.5 (6)	1	800 (3)	#2 (2)	1.881
17	3.5 (6)	2	400 (1)	#3 (3)	2.019
18	3.5 (6)	3	600 (2)	#1 (1)	1.959
$M_{1}$	5.879	11.611	11.611	11.767	*M* = 35.254
$M_{2}$	5.789	11.867	11.695	11.46	
$M_{3}$	5.847	11.776	11.948	12.027	
$M_{4}$	5.978	/	/	/	
$M_{5}$	5.932	/	/	/	
$M_{6}$	5.859	/	/	/	
*Rj*	0.189	0.256	0.337	0.567	

**Table 3:** The variance statistics from gyratory compaction tests with orthogonal array testing matrix.

Factors	Sum of Squares	Degree of Freedom	Mean Square	F	Significance
Moisture content	0.0077	5	0.0015	0.9845	0.4964
Gradation	0.0269	2	0.0134	8.6326	0.0172
Gyratory pressure	0.0103	2	0.0051	3.2976	0.1081

**Table 4:** The staged point of particle accelerations at four different positions of eighteen specimens.

Specimen Number	The Staged Point (i.e., Thresholding Number of Gyrations) of Particle Accelerations			
	Lower Center	Middle Center	Upper Center	Center Side
1	8	11	9	13
2	8	7	6	11
3	8	4	6	12
4	3	3	3	4
5	8	7	5	14
6	7	6	5	8
7	7	7	6	15
8	7	6	6	10
9	8	6	6	16
10	7	7	9	9
11	7	5	3	8
12	7	5	1	9
13	7	6	5	10
14	7	6	4	9
15	8	6	3	12
16	7	6	5	10
17	8	6	5	11
18	8	6	6	14
Average	7	6	5	11

**Table 5:** The variance statistics of particle accelerations at different positions of gyratory specimens designed by the orthogonal array method.

Position	Factor	Sum of Squares	Degree of Freedom	Mean Square	F	Significance
Upper center	Moisture content	0.00	2	0.00	0.57	0.59
	Gradation	0.01	5	0.00	1.05	0.47
	Vertical pressure	7.61	2	3.81	1589.56	0.00
Middle center	Moisture content	0.66	2	0.33	148.00	0.00
	Gradation	0.28	5	0.06	24.85	0.00
	Vertical pressure	0.12	2	0.06	28.00	0.00
Lower center	Moisture content	0.00	2	0.00	1.00	0.42
	Gradation	0.11	5	0.02	28.56	0.00
	Vertical pressure	0.00	2	0.00	1.00	0.42
Center side	Moisture content	23.80	2	11.90	1.46	0.30
	Gradation	57.01	5	11.40	1.40	0.34
	Vertical pressure	12.21	2	6.11	0.75	0.51

## Data Availability

Some or all data, models, or code that support the findings of this study are available from the corresponding author upon reasonable request.
